# Insomnia Associated With Increased Risk of Atopic Dermatitis: A Two‐Sample Mendelian Randomization Study

**DOI:** 10.1002/brb3.70512

**Published:** 2025-05-05

**Authors:** Xiuqin Ni, Xing Li, Jiaxin Li

**Affiliations:** ^1^ Department of Basic Medicine, School of Health Sciences Jiangsu Food & Pharmaceutical Science College Huai'an Jiangsu China; ^2^ Department of Neurology Hongze District People's Hospital Huai'an Jiangsu China

**Keywords:** atopic dermatitis, allergic rhinitis, allergic asthma, sleep, Mendelian randomization

## Abstract

**Background:**

The causal relationships between sleep traits and allergic diseases remain unclear. This study sought to explore their causal associations using Mendelian randomization (MR) analysis.

**Methods:**

This study utilized summary‐level data from genome‐wide association studies (GWAS) and selected genetic variants associated with sleep traits as instrumental variables (IVs). For the primary analysis, the inverse‐variance weighted (IVW) method was utilized. To further evaluate causal effects, we applied weighted median, weighted mode, and MR‐Egger regression. Sensitivity analyses, such as linkage disequilibrium score (LDSC) regression, MR‐Egger regression, Cochran's Q test, leave‐one‐out analysis, and MR‐PRESSO, were carried out to confirm result robustness.

**Results:**

IVW analysis revealed that genetically predicted insomnia was causally associated with a higher risk of atopic dermatitis (OR = 1.79, 95% CI: 1.17‐2.74, P = 0.01), and preferring an evening chronotype was causally associated with a lower risk of allergic rhinitis (IVW: OR = 0.99, 95% CI: 0.99‐1.00, P = 0.02). The LDSC analysis further identified a significant genetic correlation between insomnia and atopic dermatitis (r_g_ = 0.107, P = 0.039), but not between chronotype and allergic rhinitis (r_g_ = ‐0.036, P = 0.339). No significant connections were identified between other sleep traits and allergic diseases. The MR‐Egger intercept test did not indicate pleiotropy, except for the association with allergic asthma.

**Conclusion:**

Chronotype and insomnia were causally associated with the efficacy of sleep‐based interventions in allergic disease management.

## Graphical abstract

This study used Mendelian randomization to explore the causal relationships between sleep traits and allergic diseases. Results showed that genetically predicted insomnia increased the risk of atopic dermatitis, while an evening chronotype reduced the risk of allergic rhinitis. The analysis confirmed these associations' robustness, highlighting the potential of sleep‐based interventions in managing allergic diseases.

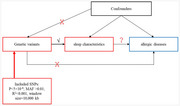

## 1 Introduction

Allergic diseases, including atopic dermatitis, allergic rhinitis, and allergic asthma, are common chronic ailments (Iordache et al. 2023; Wang et al. 2023), distinguished by an inadequate immune response to environmental allergens, leading to inflammation and symptoms that can significantly impair an individual's quality of life (Kilanowski et al. 2023). At least 20% of the global population is affected by allergic diseases, and their incidence continues to rise annually, causing both physical and mental health implications for patients and imposing significant economic burdens on society and families (Jutel et al. 2023; Mahesh et al. 2023). The etiology of allergic diseases, complex in nature, is shaped by a combination of genetic and environmental factors (Campbell et al. 2015; Zhang and Akdis 2024).

Sleep traits, as indicated by mounting evidence, may have implications for the development and progression of allergic disorders (Al Meslamani 2024; Estefan et al. 2023). Disrupted sleep patterns, including short or long sleep duration, insomnia, and altered circadian rhythms, have been linked with changes in immune function and inflammation (Akkaoui et al. 2023; Irwin 2023), which may contribute to the pathogenesis of allergic diseases (Poroyko et al. 2016). For example, increased levels of proinflammatory cytokines, such as interleukin‐6 (IL‐6) and tumor necrosis factor‐alpha (TNF‐α), associated with sleep deprivation, are implicated in the pathogenesis of allergic conditions (Chennaoui et al. 2011; Kheirandish‐Gozal and Gozal 2019). Beyond increased levels of proinflammatory cytokines, sleep disturbances may dysregulate the hypothalamic‐pituitary‐adrenal (HPA) axis, leading to altered cortisol secretion and heightened systemic inflammation (Nicolaides et al. 2020), which can exacerbate allergic responses. Additionally, sleep disturbances activate the sympathetic nervous system, increasing norepinephrine levels, which further amplify inflammatory cytokine production and disrupt immune homeostasis, contributing to allergic disease pathogenesis (Singh et al. 2024)​. Furthermore, the functioning of the immune system is closely tied to the circadian rhythm, the internal biological clock that controls the sleep‐wake cycle (Cermakian et al. 2013; Lange et al. 2010). Shifts in chronotype, reflecting alterations in the circadian rhythm (morning or evening preference), have been correlated with dysregulated inflammatory responses and an increased susceptibility to allergic diseases (Deprato et al. 2024; Fishbein et al. 2021).

Associations between different sleep traits and the risk of allergic disorders have been highlighted in prior observational studies (Liu et al. 2020; Wong et al. 2024). Nevertheless, the observational design of these studies hinders their capacity to establish causal relationships due to confounding factors (e.g., lifestyle, environmental exposures, and comorbidities) and reverse causality, as allergic diseases themselves may contribute to sleep disturbances rather than vice versa. For example, individuals with atopic dermatitis often experience itch‐induced sleep disruption, making it unclear whether poor sleep increases allergic disease risk or if preexisting allergic conditions impair sleep. Mendelian randomization (MR) addresses this limitation by employing genetic variants as instrumental variables (IVs) to infer causal relationships between an exposure and an outcome (Davey Smith and Hemani 2014; Pingault et al. 2018). Since genetic variants are randomly assigned at conception, they are not influenced by environmental factors or disease progression, reducing the risk of residual confounding. Additionally, MR estimates lifelong exposure effects, which minimizes bias from temporary fluctuations in sleep patterns and strengthens causal inference.

Up until now, there hasn't been a comprehensive inquiry into the causal relationships between a wide variety of sleep traits and the vulnerability to different allergic diseases using an MR methodology. Thus, the objective of this study was to probe the causal links between sleep traits (such as sleep duration, long sleep, short sleep, chronotype, and insomnia) and the risk of atopic dermatitis, allergic rhinitis, and allergic asthma, employing a two‐sample MR approach. By identifying potential causal relationships, this research may provide evidence for sleep‐based interventions as a modifiable strategy to reduce the burden of allergic diseases, potentially reducing disease prevalence, improving quality of life, and lowering healthcare costs.

## 2 Materials and Methods

### 2.1 Study Design

**FIGURE 1:**
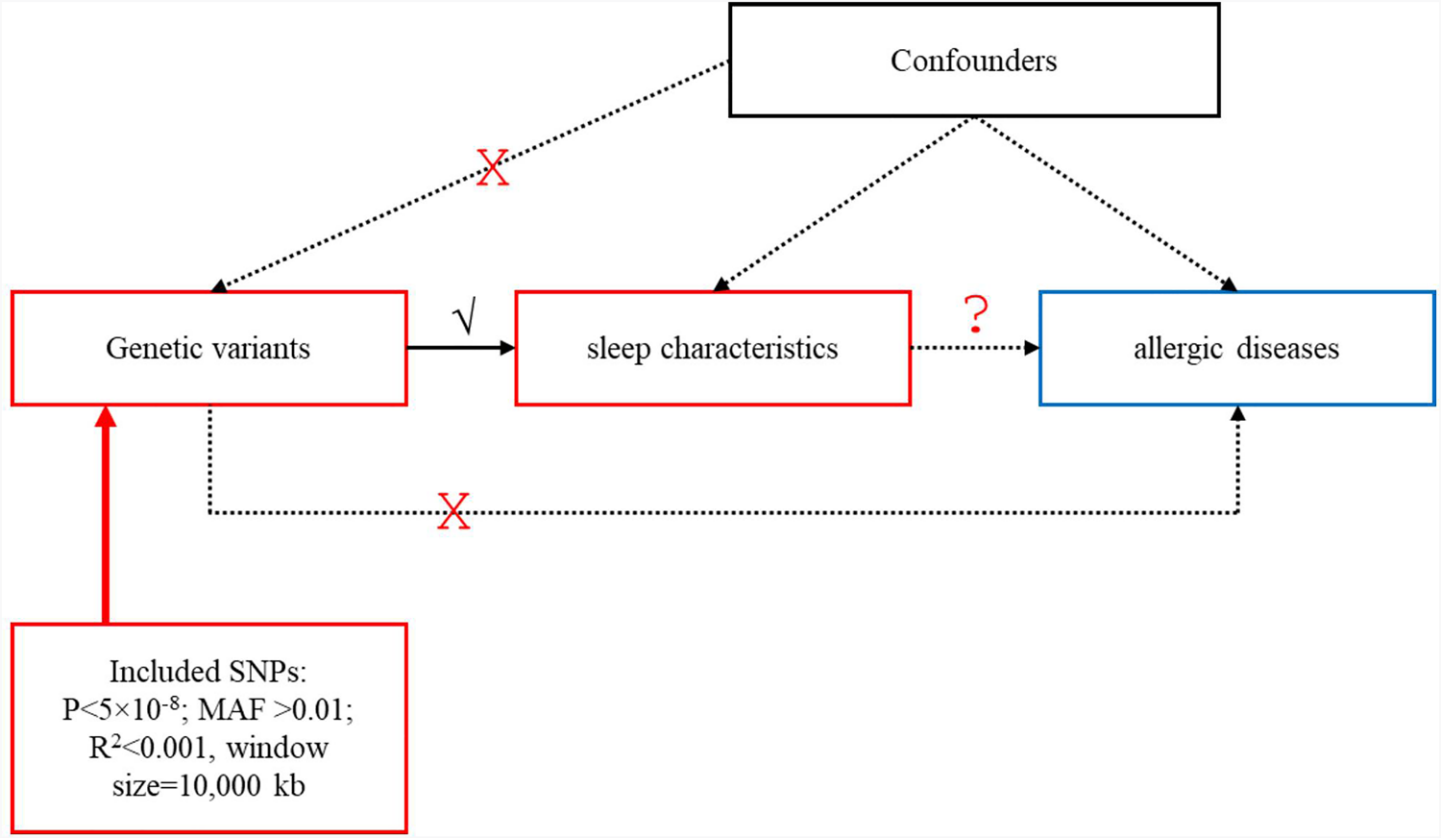
Description of the study design in this MR study.

Utilizing genetic variants strongly associated with exposure as IVs, we conducted MR to ascertain the causal relationship between exposure (such as sleep duration, long sleep, short sleep, chronotype, and insomnia) and outcomes (namely, atopic dermatitis, allergic rhinitis, and allergic asthma). Regarding genetic variation, the MR design relies on three crucial assumptions (Richmond and Smith 2022): (1) Strong association between genetic variants and the exposure; (2) Independence of genetic variants from potential confounding factors; (3) Genetic variants influencing outcomes exclusively through exposure factors (Figure 1).

### 2.2 Data Sources

Obtained from three separate self‐reported studies conducted by the United Kingdom Biobank project between 2006 and 2010, the summary‐level data from genome‐wide association studies (GWAS) on genetic associations with sleep traits were utilized. For the MR analysis, 78 SNPs associated with continuous sleep duration (measured as self‐reported habitual sleep duration in hours), 26 SNPs associated with short sleep duration (< 7 hours per night), and 10 SNPs associated with long sleep duration (≥ 9 hours per night) were included (Dashti et al. 2019). In addition, 41 SNPs related to insomnia symptoms were included, with insomnia assessed based on self‐reported difficulty falling or staying asleep (Lane et al. 2019). Additionally, 153 SNPs associated with chronotype (Jones et al. 2019) were integrated. Chronotype genetic associations were represented on an integer scale, capturing an individual's preference for morningness or eveningness. The scale ranged from 2 (“Definitely a morning person”), 1 (“More a morning than evening person”), 0 (“Do not know”), ‐1 (“More an evening than morning person”), and ‐2 (“Definitely an evening person”). This scale reflects a continuous spectrum in which higher values indicate a preference for waking and sleeping earlier (morningness), and lower values indicate a shift toward later sleep and wake times (eveningness). Individuals categorized as 2 or 1 are more active in the morning, whereas those with ‐1 or ‐2 prefer later activity, with 0 representing those who are uncertain about their chronotype. The GWAS dataset for atopic dermatitis (ID: ebi‐a‐GCST90027161, sample size: 796,661) contained 16,121,213 SNPs (Sliz et al. 2022). The datasets for allergic rhinitis (ID: ebi‐a‐GCST90038664, sample size: 484,598) were utilized (Dönertaş et al. 2021). Genetic IVs for allergic asthma, obtained from GWAS conducted within the FinnGen database, encompass a total of 170,583 individuals of European origin, with 4,859 cases and 135,449 controls. For further detailed information about FinnGen, please visit its official website: https://www.finngen.fi/en. Additional details can be found in Table S1.

The present study employs GWAS data sourced from publicly available databases, which were previously ethically approved in their respective original investigations.

### 2.3 Selection of IVs

IVs were selected based on genome‐wide significance (p < 5 × 10⁻^8^) and linkage disequilibrium (LD) clumping parameters (kb = 10,000, r^2^ = 0.01) to ensure independence. To strengthen biological relevance, SNPs previously associated with sleep traits in GWAS were prioritized. To minimize weak instrument bias and improve statistical power, only SNPs with F‐statistics >10 were retained (Burgess et al. 2011).

### 2.4 MR Analysis

MR analysis was conducted using the TwoSampleMR package (version 4.3.1) (Yavorska and Burgess 2017). The primary method was the inverse‐variance‐weighted (IVW) model, which provides the highest statistical power when pleiotropy is balanced (Lin et al. 2021). To ensure the reliability of the findings, additional MR methods were applied. The weighted median estimator is preferred when up to 50% of SNPs are invalid due to pleiotropy. MR‐Egger regression is used when directional pleiotropy is suspected, as it can detect and adjust for such bias. The weighted mode method is useful when most valid instruments cluster around a consistent causal estimate. These complementary approaches help evaluate the robustness of the IVW results and account for potential violations of MR assumptions (Boehm and Zhou 2022). A significance threshold of p < 0.05 was applied to determine causal effects.

### 2.5 Sensitivity Analysis

To ensure the validity of the genetic instruments, linkage disequilibrium score (LDSC) regression was used to estimate SNP‐based heritability for sleep traits and allergic diseases. SNPs with high imputation quality (INFO score >0.9) and minor allele frequency (MAF) >0.01 were retained by filtering against HapMap3 SNPs. The SNP‐based heritability of sleep traits and allergic diseases was estimated, and the baseline LD model was applied to control for confounders (Bulik‐Sullivan et al. 2015). Then, genetic correlations between the conditions were calculated to infer causal relationships. The analysis was performed using the LDSC software (v1.0.1).

Cochran's Q test, a pleiotropy test, and a leave‐one‐out sensitivity test, were conducted to assess significantly associated sleep characteristic SNPs. In case the pleiotropy test indicated the existence of pleiotropy (p < 0.05), MR‐PRESSO was utilized to identify and correct potential outliers (Bowden et al. 2015). The final step involved performing a leave‐one‐out sensitivity analysis to determine if the exclusion of any individual SNP yielded significant results.

## 3 Results

### 3.1 IVs Selection

When this study performed MR analysis using sleep duration, long sleep, short sleep, chronotype, and insomnia as exposures, it selected 70, 10, 26, 152, and 41 related IVs, respectively. The average F statistic values were 40.51, 39.89, 37.21, 48.07, and 42.89. The minimum F statistic values recorded were 25.69, 29.89, 29.9, 28.17, and 29.24, while the maximum values were 220.87, 52.98, 77.04, 209.39, and 181.16. Additionally, there were 12, 3, 0, 19, and 7 SNPs that did not match the summary data information, respectively. Details regarding the number of SNPs are provided in Table S2.

### 3.2 MR Analysis

**FIGURE 2:**
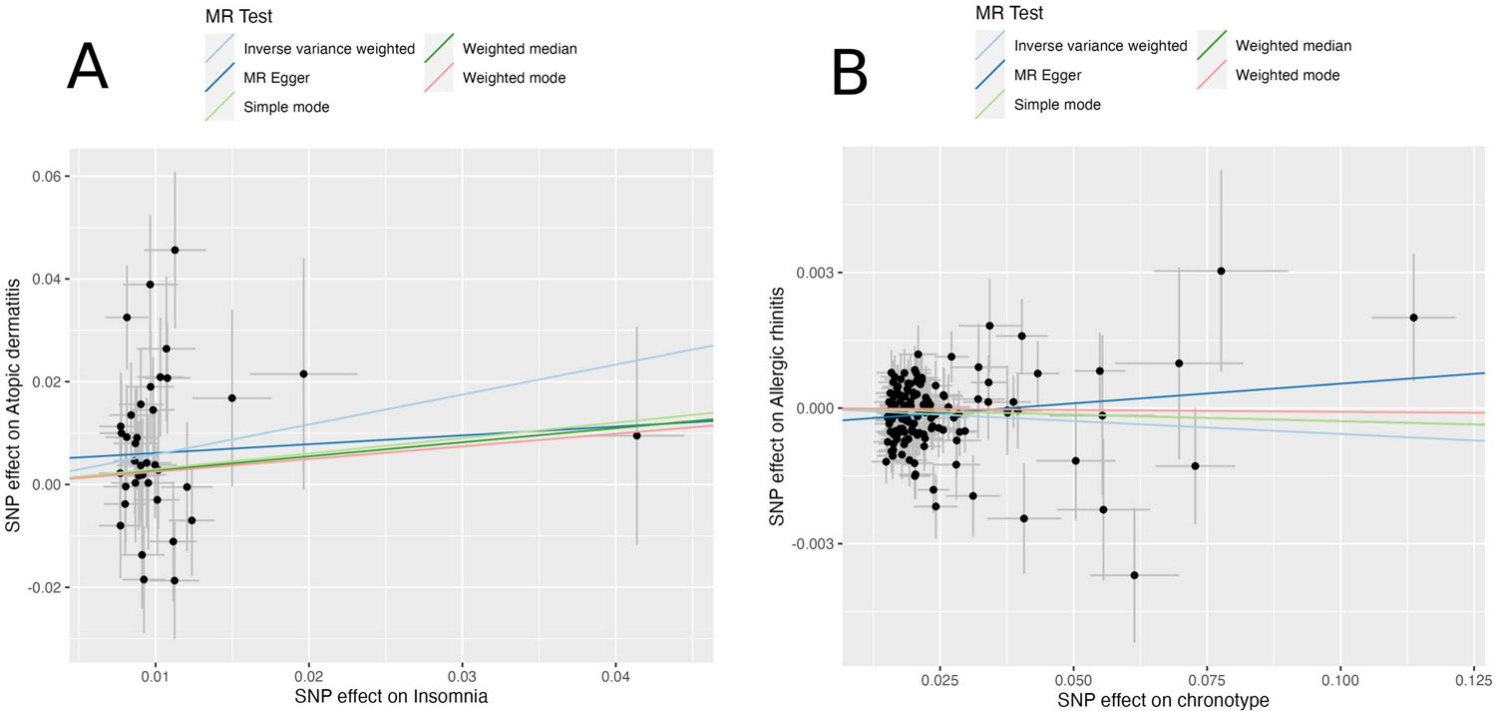
Scatter plots showing the causal effects of sleep traits on allergic diseases. (A) Single nucleotide polymorphism (SNP) effects of insomnia on atopic dermatitis. (B) SNP effects of chronotype on allergic rhinitis. Each black dot represents an SNP. Horizontal and vertical error bars indicate standard errors. The lines correspond to different Mendelian randomization methods: inverse variance weighted (light blue), MR Egger (dark blue), weighted median (green), weighted mode (red), and simple mode (light green).

The study revealed an association between genetically predetermined sleep traits and susceptibility to certain allergic conditions. Specifically, individuals with a genetically inferred evening chronotype demonstrated a marginally reduced risk of developing allergic rhinitis (IVW OR = 0.99; 95% CI = 0.99‐1.00, p = 0.02). While this association is statistically significant, the OR of 0.99 indicates a very small effect, suggesting that the practical significance of this finding may be limited. Conversely, genetic predisposition to insomnia was found to be significantly correlated with an elevated risk of atopic dermatitis (IVW OR = 1.79, 95% CI = 1.17‐2.74, p = 0.01), highlighting a potential risk factor. A comprehensive summary of these MR analysis outcomes is presented in Table 1, offering a detailed insight into the genetic interplay between sleep traits and allergic disease risks. To facilitate intuitive understanding, the estimated effect sizes of SNPs linked to sleep traits on the prevalence of allergic diseases were visually depicted through a scatter plot (Figure 2 and Figure S1). To validate the significant results from IVW analysis, LDSC regression was further performed to assess their genetic correlations. The LDSC analysis identified a significant genetic correlation between insomnia and atopic dermatitis (r_g_ = 0.107, p = 0.039). The SNP‐based heritability estimates were 0.008 for sleep traits and 0.119 for allergic diseases. However, no genetic correlation was observed between chronotype and allergic rhinitis (r_g_ = ‐0.036, p = 0.339) (Table 2).

**TABLE 1:** Association between sleep traits and allergic diseases based on Mendelian randomization analysis.

Exposure	Outcome	Number of SNPs	Methods	OR (95% CI)	P
Sleep duration	Atopic dermatitis	67	Inverse variance weighted	0.98 (0.81 ‐ 1.18)	0.80
	Allergic rhinitis	67	Inverse variance weighted	0.99 (0.98 ‐ 1)	0.17
	Allergic asthma	67	Inverse variance weighted	0.78 (0.5 ‐ 1.22)	0.27
Long sleep	Atopic dermatitis	8	Inverse variance weighted	0.46 (0.03 ‐ 6.69)	0.57
	Allergic rhinitis	9	Inverse variance weighted	1.01 (0.93 ‐ 1.11)	0.74
	Allergic asthma	8	Inverse variance weighted	0.08 (0 ‐ 17.7)	0.36
Short sleep	Atopic dermatitis	24	Inverse variance weighted	1.72 (0.84 ‐ 3.49)	0.14
	Allergic rhinitis	23	Inverse variance weighted	0.99 (0.95 ‐ 1.03)	0.75
	Allergic asthma	24	Inverse variance weighted	2.73 (0.41 ‐ 18.39)	0.30
Chronotype	Atopic dermatitis	147	Inverse variance weighted	0.93 (0.85 ‐ 1.03)	0.15
	Allergic rhinitis	147	Inverse variance weighted	0.99 (0.99 ‐ 1)	0.02
	Allergic asthma	145	Inverse variance weighted	1.15 (0.93 ‐ 1.41)	0.20
Insomnia	Atopic dermatitis	38	Inverse variance weighted	1.79 (1.17 ‐ 2.74)	0.01
	Allergic rhinitis	38	Inverse variance weighted	1 (0.98 ‐ 1.02)	0.72
	Allergic asthma	38	Inverse variance weighted	1.39 (0.64 ‐ 2.99)	0.40

**TABLE 2:** Genetic correlation estimates between sleep traits and allergic diseases using LDSC regression.

Trait 1	Trait 2	*r_g_*	*r_g_* _SE	P	Heritability of Trait 1 (Z score ± SE)	Heritability of Trait 2 (Z score ± SE)
Atopic dermatitis	Insomnia	0.107	0.052	0.039	0.008 (0.002)	0.119 (0.006)
Allergic rhinitis	Chronotype	−0.036	0.038	0.339	0.025 (0.003)	0.131 (0.008)

### 3.3 Sensitivity Analysis

The heterogeneity tests (measured by the Q‐statistic under the IVW method) revealed moderate to high levels of heterogeneity for several exposure‐outcome pairs, such as sleep duration with allergic rhinitis (Q = 105.62, p < 0.01) and allergic asthma (Q = 98.88, p < 0.01), indicating variability among the instrumental variables. However, the MR‐Egger intercept test, used to assess potential pleiotropy, did not yield statistically significant evidence for pleiotropic effects for the majority of outcomes, except for allergic asthma in relation to sleep duration (p = 0.037) and long sleep (p = 0.031), as well as allergic rhinitis in relation to chronotype (p = 0.042), suggesting that directional bias due to pleiotropy might be minimal (Table S3).

In the case of short sleep and chronotype related to allergic rhinitis, the MR‐PRESSO test results (Table S4) highlighted the presence of outliers, which could distort the overall estimate. Upon correcting for these outliers, the associations retained significance, suggesting that the initial observations were not entirely driven by these outliers. For instance, the association between short sleep and allergic rhinitis was slightly attenuated but still non‐significant after correction (OR = 0.99, 95% CI = 0.95‐1.03), with one outlier identified and a global P‐value indicating some distortion (p < 0.001, distortion p = 0.088). Similarly, the chronotype‐outcome link showed a similar pattern with one outlier, yet the corrected association remained marginal (OR = 0.99, 95% CI: 0.990‐0.999), signifying a minor influence from the outlier and robustness in the overall finding (global p < 0.001, distortion p = 0.627).

As part of an additional sensitivity assessment, a “leave‐one‐out” procedure was undertaken during the MR analysis. The leave‐one‐out plots are shown in Figures S2 and S3, demonstrating consistency of estimates with primary IVW results. This underscores the robustness of findings across analyses, regardless of the presence or absence of individual IVs.

Overall, these sensitivity analyses underscore the complexity inherent in examining genetic influences on sleep patterns and their relationship with allergic diseases. While some degree of heterogeneity and potential outlier effects were detected, the core findings generally remained consistent post‐correction, reinforcing the validity of the reported associations.

## 4 Discussion

This study, utilizing a two‐sample MR approach, thoroughly explored the causal connections between a spectrum of sleep traits and the vulnerability to three common allergic conditions: atopic dermatitis, allergic rhinitis, and allergic asthma. The primary outcomes of this investigation include: (1) genetically forecasted chronotype exhibited an association with decreased allergic rhinitis risk, and (2) genetically forecasted insomnia displayed an association with increased atopic dermatitis risk.

This study identified several key SNPs contributing to the observed association between insomnia and atopic dermatitis, including rs6664467 (*MRPL9‐TDRKH*), rs17669584 (*SMURF2P‐KRT17P3*), and rs11635495 (*IQCH‐AS1*). *MRPL9* encodes a mitochondrial ribosomal protein L9, which plays a role in cellular energy homeostasis and immune cell activation (Branco et al. 2018). Mitochondrial dysfunction can lead to increased production of reactive oxygen species, resulting in oxidative stress that damages cellular components, disrupts skin barrier integrity, and triggers inflammatory pathways, potentially contributing to the pathogenesis of atopic dermatitis (Natarelli et al. 2024). Although *MRPL9* has not been previously linked to insomnia, mitochondrial dysfunction and oxidative stress are known to impair neuronal function and disrupt circadian rhythms, which could contribute to sleep disturbances (Heyat et al. 2022), providing a potential mechanistic basis for our observed genetic association. SMURF2, an E3 ubiquitin ligase, regulates TGF‐β signaling, a pathway involved in immune homeostasis, fibrosis, and epithelial barrier integrity (Bai and Ying 2020). Dysregulated *SMURF2* expression has been implicated in inflammatory skin diseases such as psoriasis and vitiligo (Liu et al. 2016), likely through alterations in cytokine homeostasis, including IL‐6, IL‐10, and TGF‐β1, which are also central to atopic dermatitis pathophysiology (Park et al. 2020). Although a direct connection between TGF‐β signaling and sleep disturbances remains unestablished, its regulatory role in neuroinflammation and immune function suggests potential relevance to sleep regulation (Zielinski and Gibbons 2022). *SMURF2* may act as a molecular bridge between immune dysfunction, inflammatory skin disorders, and disrupted sleep patterns, warranting further investigation into its involvement in atopic dermatitis‐associated sleep disturbances. *IQCH‐AS1*, a long non‐coding RNA, is involved in gene regulation by acting as a competing endogenous RNA that modulates microRNA activity (Fei et al. 2023). Although *IQCH‐AS1* has not been directly linked to insomnia, the identification of *IQCH* in both insomnia and asthma GWAS (Kim et al. 2021) suggests its potential role in immune‐related neurophysiological processes. Given the well‐established immunological overlap between asthma and atopic dermatitis, this finding supports the hypothesis that *IQCH‐AS1* contributes to inflammatory responses and disrupted sleep regulation in atopic dermatitis (Yaneva and Darlenski 2021). This study is the first to identify specific SNPs within *MRPL9, SMURF2*, and *IQCH‐AS1* that contribute to the genetic association between insomnia and atopic dermatitis. Future studies should validate the causal role of these SNPs through experimental and longitudinal approaches to clarify their contribution to the shared pathophysiology of both disorders.

The observed relationship between chronotype and allergic rhinitis aligns with the strong link between circadian rhythms and sleep traits. Chronotype, representing an individual's preference for morning or evening activities, is a manifestation of their circadian rhythms (Montaruli et al. 2021). Numerous physiological processes, including the sleep‐wake cycle, hormone secretion, and immune function, are regulated by circadian rhythms (Sarisozen et al. 2023). Disturbances in circadian rhythms can result in sleep disorders like insomnia and sleep fragmentation, potentially exacerbating the onset of allergic conditions (Fishbein et al. 2021; Zisapel 2007). Regulation of immune response genes is managed by the circadian clock, and if this system is disrupted, it can exacerbate inflammation and allergic symptoms (Cheng et al. 2022; Nakao 2020). Altered circadian rhythms, for instance, can disturb the production of cortisol, an essential anti‐inflammatory hormone, thereby enhancing airway inflammation and exacerbating symptoms of allergic rhinitis (Comas et al. 2017; D'Elia et al. 2022). In addition, long‐term sleep disturbances associated with disruptions in circadian rhythms have been connected to oxidative stress and modifications in cytokine production, ultimately fueling allergic responses (Chang and Chiang 2016; Pham et al. 2021). Similarly, a recent MR study revealed the causal associations between allergic rhinitis and sleep disorders, potentially mediated by inflammatory pathways (Lin et al. 2024). However, the causal associations between chronotype and allergic rhinitis were not captured in further LDSC analysis. The null significance suggests that other factors, such as environmental exposure and behavior, may play a role in the interaction between chronotype and allergic diseases (Prescott 2013).

The observed inverse association between evening chronotype and allergic rhinitis contrasts with prior observational studies linking evening chronotype to adverse health outcomes (Makarem et al. 2020). This discrepancy may stem from differences in genetic influences versus environmental confounders in observational studies, as well as potential distinct immune mechanisms underlying rhinitis and dermatitis, such as the dominance of IgE‐mediated Th2 responses in allergic rhinitis (Zhang et al. 2022) versus the mixed Th1/Th2 inflammation and skin barrier dysfunction characteristic of atopic dermatitis (Meng et al. 2021). Specific genetic variants associated with the evening chronotype could modulate immune function in a protective manner against allergic rhinitis, but not against other allergic conditions, possibly through differential effects on mucosal immunity, IgE regulation, or circadian control of inflammatory responses, warranting further investigation. Additionally, population characteristics, including lifestyle patterns and exposure to allergens, may modify the relationship between chronotype and allergic diseases. Hence, further research is required to verify the causality between chronotype and allergic diseases and elucidate the underlying pathways.

Our findings suggest that optimizing sleep patterns and circadian alignment is relevant for managing allergic conditions. Given the association between insomnia and atopic dermatitis, interventions that improve sleep quality, such as cognitive behavioral therapy for insomnia, maintaining consistent sleep schedules, and reducing nighttime light exposure, could be explored as adjunctive approaches in atopic dermatitis management. Additionally, understanding how chronotype influences allergic rhinitis risk may help tailor lifestyle recommendations, such as avoiding outdoor exposure during peak pollen hours (typically early morning and late evening), using air purifiers at night to reduce indoor allergen load, and adjusting antihistamine or corticosteroid intake to align with circadian fluctuations in immune response. Future studies should assess whether implementing these sleep‐focused and exposure‐based strategies can modify allergic disease trajectories.

The MR approach utilized in this study aims to overcome limitations and offer more robust evidence for causal relationships. By employing genetic variants as IVs, MR can mitigate the impact of confounding and reverse causation, which are common challenges in observational studies. However, MR analysis may still be influenced by horizontal pleiotropy, where the genetic variants used as IVs exert effects on the outcome through pathways unrelated to the exposure of interest (Hemani et al. 2018; Verbanck et al. 2018). To address this concern, sensitivity analyses, including MR‐Egger regression and the MR‐PRESSO method, were conducted to evaluate the potential influence of pleiotropy and outliers on the MR findings.

MR‐Egger intercept tests suggested potential pleiotropy for allergic asthma in relation to sleep duration (p = 0.037) and long sleep (p = 0.031). The presence of pleiotropy in allergic asthma could reflect the complex immune and inflammatory pathways involved, as asthma is influenced by both systemic and airway‐specific inflammatory processes that may interact with sleep disturbances differently compared to atopic dermatitis and allergic rhinitis. In contrast, the absence of pleiotropy in other outcomes suggests that genetic instruments for sleep traits predominantly affect these conditions through pathways directly linked to immune regulation and circadian rhythm alignment, rather than via horizontal pleiotropy. Furthermore, the consistency of causal estimates across multiple MR methods supports the robustness of our findings despite the observed pleiotropy in allergic asthma. However, caution is warranted when interpreting the asthma‐related results, and future studies should further investigate the potential pleiotropic pathways linking sleep and asthma risk.

Notably, non‐significant associations between other sleep traits, such as sleep duration, and allergic diseases did not imply no causal relationship. The MR approach depends on the availability of reliable and valid genetic instruments, and the ability to detect causal effects might be constrained by the sample size and the magnitude of the genetic associations. Furthermore, the intricate interplay among various sleep traits and their potential interactions with other environmental and genetic factors could contribute to the onset of allergic disorders, a complexity that this study didn't entirely encompass.

Nevertheless, there are several limitations to acknowledge. Firstly, the study relied on summary‐level data from GWAS, which limits the exploration of individual‐level interactions and gene‐environment effects. Second, while the MR approach helps strengthen causal inference by reducing confounding and reverse causation, it relies on key assumptions, including the absence of horizontal pleiotropy, which may still be present despite sensitivity analyses. Third, the generalizability of the findings may be restricted, as the GWAS data predominantly originated from individuals of European descent. Future population‐based studies with diverse ancestries are needed to validate these results. Lastly, this study did not investigate temporal relationships or dynamic changes in sleep traits and allergic disease risk over time, highlighting the need for further longitudinal research.

## 5 Conclusion

In conclusion, this MR research contributes evidence indicating that chronotype and insomnia may causally affect the risk of allergic rhinitis and atopic dermatitis, respectively. These findings raise the prospect of sleep traits serving as potential targets for preventing and managing certain allergic conditions. More research is warranted to clarify the underlying biological mechanisms and assess the potential for incorporating sleep‐based interventions into allergic disease management.

## Author Contributions

**Xiuqin Ni**: conceptualization, investigation, writing – original draft, writing – review and editing, methodology, formal analysis, data curation. **Xing Li**: conceptualization, investigation, writing – original draft, methodology, writing – review and editing, formal analysis, data curation. **Jiaxin Li**: conceptualization, formal analysis, writing – review and editing.

## Ethics Statement

Xiuqin Ni and Xing Li carried out the studies, participated in collecting data, and drafted the manuscript. Xiuqin Ni and Jiaxin Li performed the statistical analysis and participated in its design. Xiuqin Ni and Xing Li participated in acquisition, analysis, or interpretation of data and draft the manuscript. All authors read and approved the final manuscript.

## Conflicts of Interest

The authors declare no conflicts of interest.

### Peer Review

The peer review history for this article is available at https://publons.com/publon/10.1002/brb3.70512

## Supporting information




**Figure S1. Scatter plots showing the causal effects of sleep traits on allergic diseases**. Each panel represents the relationship between a specific sleep trait (x‐axis: SNP effect on sleep trait) and an allergic disease (y‐axis: SNP effect on allergic disease). (A–D) Effects of sleep duration, long sleep, short sleep, and chronotype on atopic dermatitis. (E–H) Effects of sleep duration, long sleep, short sleep, and insomnia on allergic rhinitis. (I–M) Effects of sleep duration, long sleep, short sleep, chronotype, and insomnia on allergic asthma. Each black dot represents a single nucleotide polymorphism (SNP). Horizontal and vertical error bars indicate standard errors. The lines correspond to different Mendelian randomization methods: inverse variance weighted (light blue), MR Egger (dark blue), weighted median (green), weighted mode (red), and simple mode (light green).

**Figure S2**. **Leave‐one‐out sensitivity analysis of sleep traits on allergic diseases (comprehensive overview**). Each panel represents the results of the leave‐one‐out analysis, in which individual SNPs were iteratively removed to assess their influence on the overall causal estimate. The x‐axis shows the effect estimate, and the y‐axis lists the SNPs used as instrumental variables. (A–D) Effects of sleep duration, long sleep, short sleep, and chronotype on atopic dermatitis. (E–H) Effects of sleep duration, long sleep, short sleep, and insomnia on allergic rhinitis. (I–M) Effects of sleep duration, long sleep, short sleep, chronotype, and insomnia on allergic asthma. Each black dot represents the causal estimate after removing one SNP. Horizontal lines indicate confidence intervals. The red line represents the overall Mendelian randomization estimate using all SNPs.

**Figure S3**. **Leave‐one‐out sensitivity analysis of insomnia and chronotype on allergic diseases (significant findings)**. Each panel represents the results of the leave‐one‐out analysis, in which individual SNPs were iteratively removed to assess their influence on the overall causal estimate. The x‐axis shows the effect estimate, and the y‐axis lists the SNPs used as instrumental variables. (A) Insomnia on atopic dermatitis. (B) Chronotype on allergic rhinitis. Each black dot represents the causal estimate after removing one SNP. Horizontal lines indicate confidence intervals. The red line represents the overall Mendelian randomization estimate using all SNPs.

**Table S1**. Summary of GWAS data for sleep traits and allergic diseases.

**Table S2**. Instrumental variable selection for Mendelian randomization analysis of sleep traits and allergic diseases.

**Table S3**. Heterogeneity and pleiotropy tests for instrumental variables in Mendelian randomization analysis.

**Table S4**. MR‐PRESSO test results for detecting and correcting outlier effects in Mendelian randomization analysis.

## Data Availability

All data generated or analyzed during this study are included in this article and supplementary information files.
