# Supplementary figures and images for: Insomnia Associated With Increased Risk of Atopic Dermatitis: A Two‐Sample Mendelian Randomization Study
Source: Brain Behav. 2025 May 5;15(5):e70512. doi: 10.1002/brb3.70512 (PMC12050649; doi:10.1002/brb3.70512)

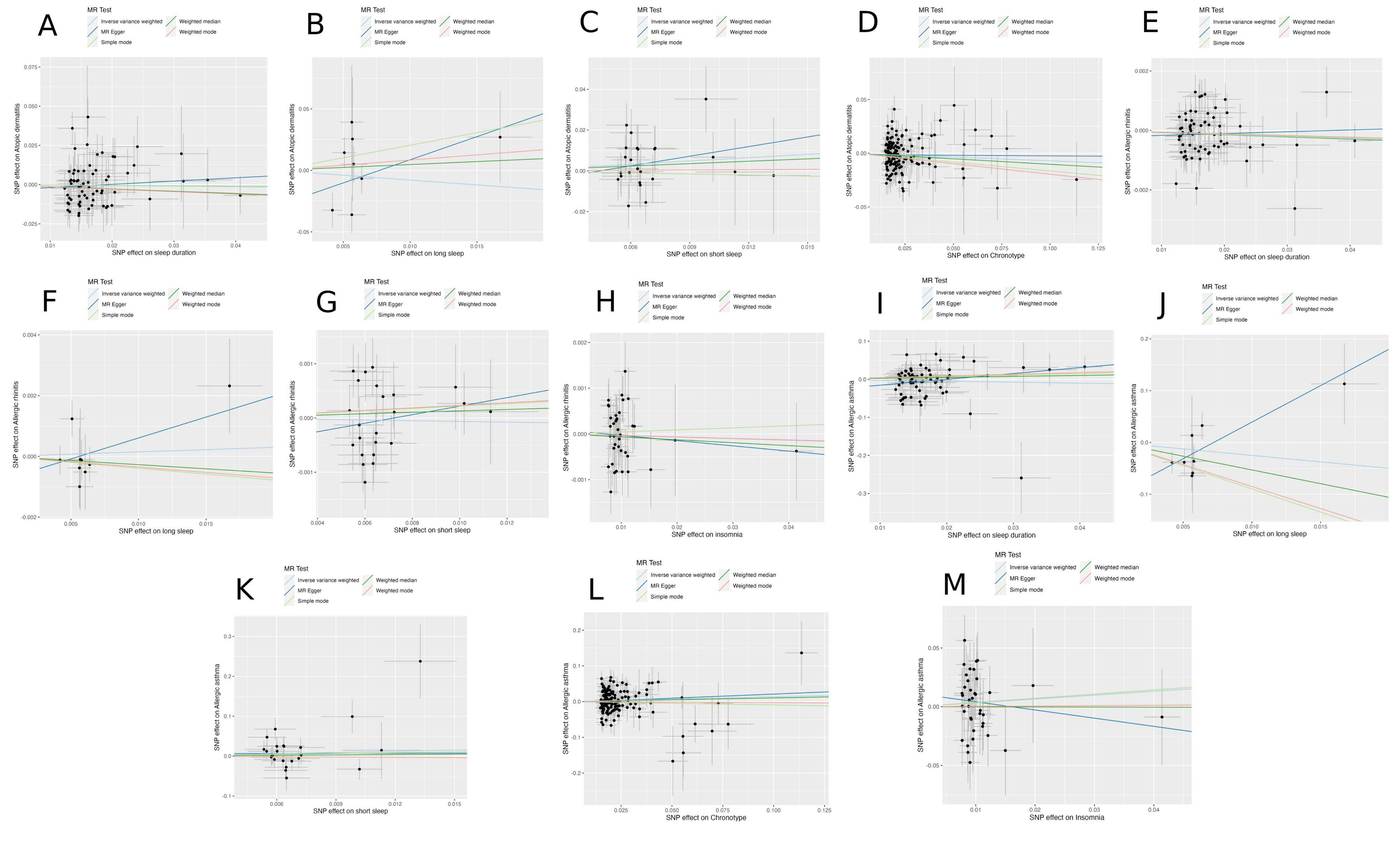

Supplement: Supplementary file 1 — Figure S1. Scatter plots showing the causal effects of sleep traits on allergic diseases. Each panel represents the relationship between a specific sleep trait (x‐axis: SNP effect on sleep trait) and an allergic disease (y‐axis: SNP effect on allergic disease). (A–D) Effects of sleep duration, long sleep, short sleep, and chronotype on atopic dermatitis. (E–H) Effects of sleep duration, long sleep, short sleep, and insomnia on allergic rhinitis. (I–M) Effects of sleep duration, long sleep, short sleep, chronotype, and insomnia on allergic asthma. Each black dot represents a single nucleotide polymorphism (SNP). Horizontal and vertical error bars indicate standard errors. The lines correspond to different Mendelian randomization methods: inverse variance weighted (light blue), MR Egger (dark blue), weighted median (green), weighted mode (red), and simple mode (light green). [file BRB3-15-e70512-s004.jpg]

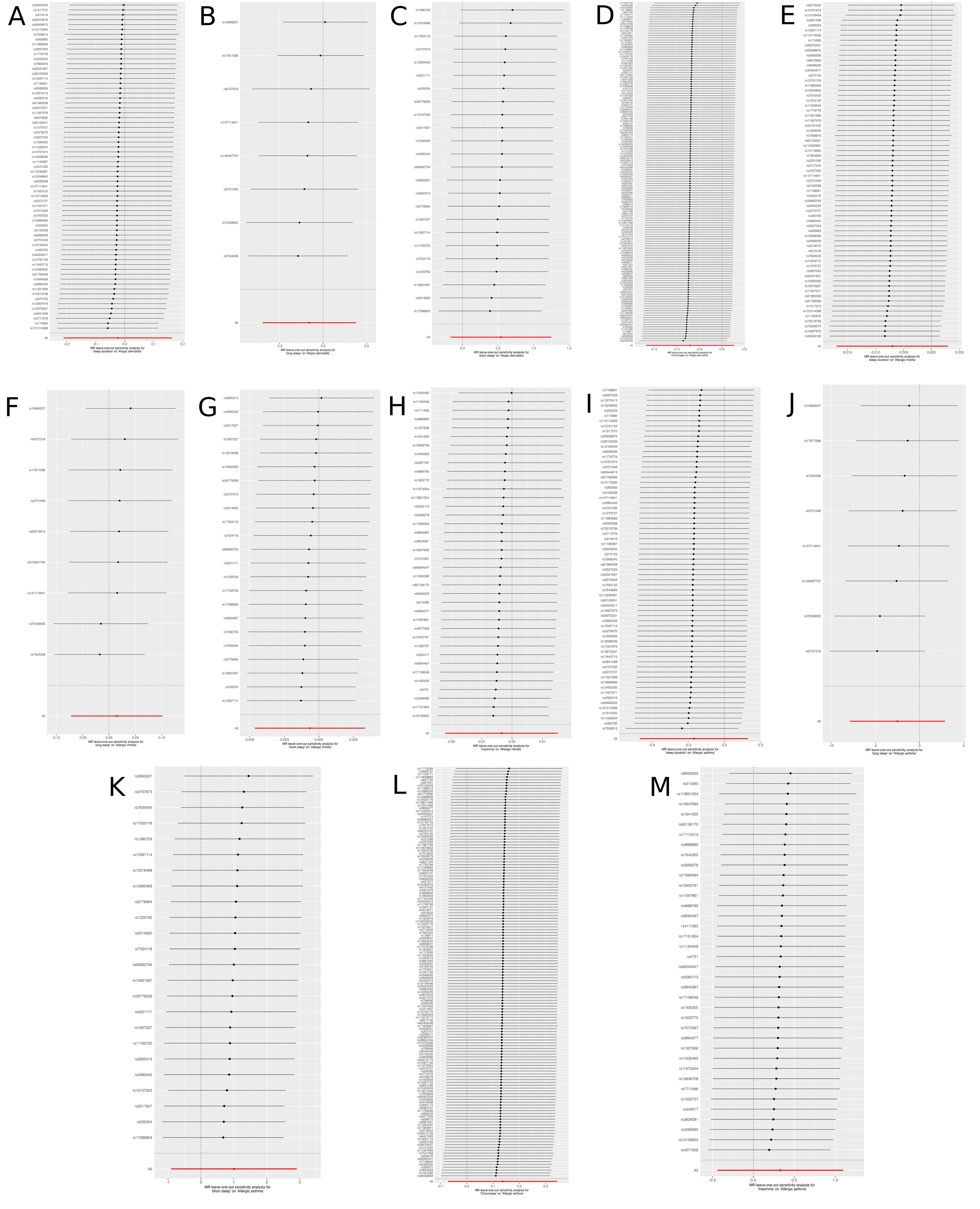

Supplement: Supplementary file 2 — Figure S2. Leave‐one‐out sensitivity analysis of sleep traits on allergic diseases (comprehensive overview). Each panel represents the results of the leave‐one‐out analysis, in which individual SNPs were iteratively removed to assess their influence on the overall causal estimate. The x‐axis shows the effect estimate, and the y‐axis lists the SNPs used as instrumental variables. (A–D) Effects of sleep duration, long sleep, short sleep, and chronotype on atopic dermatitis. (E–H) Effects of sleep duration, long sleep, short sleep, and insomnia on allergic rhinitis. (I–M) Effects of sleep duration, long sleep, short sleep, chronotype, and insomnia on allergic asthma. Each black dot represents the causal estimate after removing one SNP. Horizontal lines indicate confidence intervals. The red line represents the overall Mendelian randomization estimate using all SNPs. [file BRB3-15-e70512-s007.jpg]

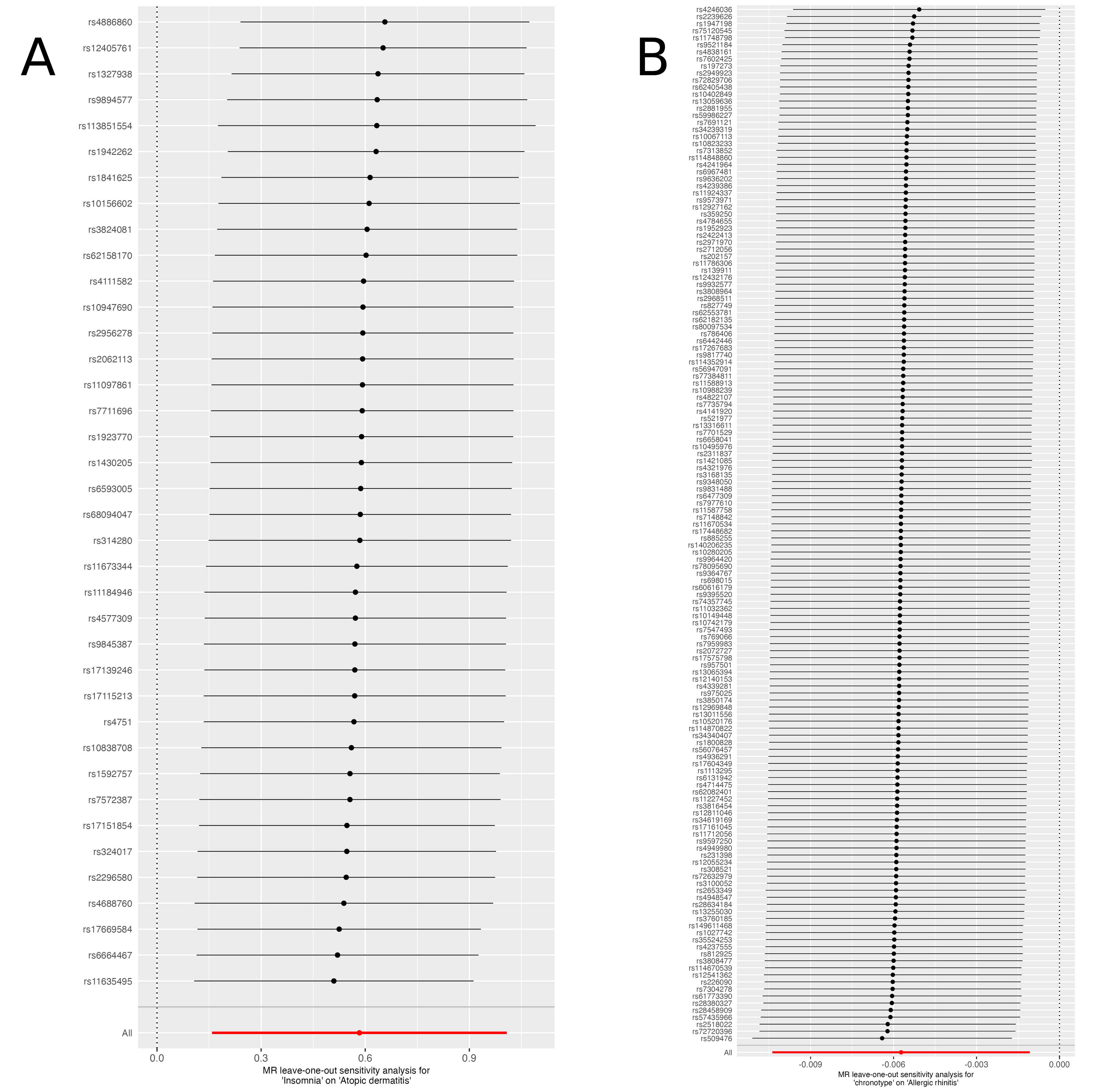

Supplement: Supplementary file 3 — Figure S3. Leave‐one‐out sensitivity analysis of insomnia and chronotype on allergic diseases (significant findings). Each panel represents the results of the leave‐one‐out analysis, in which individual SNPs were iteratively removed to assess their influence on the overall causal estimate. The x‐axis shows the effect estimate, and the y‐axis lists the SNPs used as instrumental variables. (A) Insomnia on atopic dermatitis. (B) Chronotype on allergic rhinitis. Each black dot represents the causal estimate after removing one SNP. Horizontal lines indicate confidence intervals. The red line represents the overall Mendelian randomization estimate using all SNPs. [file BRB3-15-e70512-s006.jpg]
