# Supplementary material for: Insomnia Associated With Increased Risk of Atopic Dermatitis: A Two‐Sample Mendelian Randomization Study
Source: Brain Behav. 2025 May 5;15(5):e70512. doi: 10.1002/brb3.70512 (PMC12050649; doi:10.1002/brb3.70512)
Supplement: Supplementary file 4 — Table S1. Summary of GWAS data for sleep traits and allergic diseases. [file BRB3-15-e70512-s001.docx]

**Table S1. Detailed information for the GWAS data.**

| **Trait** | **GWAS ID** | **Number of Participants** | **Number of SNPs** | **Population** |
| --- | --- | --- | --- | --- |
| Sleep duration | GCST007561 | 446,118 | 72 | European |
| Long sleep | GCST007560 | 34,184/305,742 | 8 | European |
| Short sleep | GCST007559 | 106,192/305,742 | 27 | European |
| Chronotype | GCST007576 | 252,287/150,908 | 440 | European |
| Insomnia | GCST007387 | 129,270/108,357 | 49 | European |
