# Supplementary material for: Insomnia Associated With Increased Risk of Atopic Dermatitis: A Two‐Sample Mendelian Randomization Study
Source: Brain Behav. 2025 May 5;15(5):e70512. doi: 10.1002/brb3.70512 (PMC12050649; doi:10.1002/brb3.70512)
Supplement: Supplementary file 6 — Table S3. Heterogeneity and pleiotropy tests for instrumental variables in Mendelian randomization analysis. [file BRB3-15-e70512-s005.docx]

**Table S3. Results of heterogeneity test and pleiotropy test of instrumental variables.**

| **Exposure** | **Outcome** | **Heterogeneity** | | **Pleiotropy** | |
| --- | --- | --- | --- | --- | --- |
|  |  | **Q statistic (IVW)** | ***P* value** | **MR-Egger Intercept** | ***P* value** |
| Sleep duration | Atopic dermatitis | 78.71 | 0.14 | -0.004 | 0.522 |
|  | Allergic rhinitis | 105.62 | <0.01 | <0.001 | 0.489 |
|  | Allergic asthma | 98.88 | <0.01 | -0.031 | 0.037 |
| Long sleep | Atopic dermatitis | 13.79 | 0.06 | -0.029 | 0.304 |
|  | Allergic rhinitis | 9.32 | 0.32 | >-0.001 | 0.372 |
|  | Allergic asthma | 13.63 | 0.06 | -0.102 | 0.031 |
| Short sleep | Atopic dermatitis | 20.7 | 0.6 | -0.007 | 0.575 |
|  | Allergic rhinitis | 31.93 | 0.08 | >-0.001 | 0.452 |
|  | Allergic asthma | 35.23 | 0.05 | 0.006 | 0.869 |
| Chronotype | Atopic dermatitis | 173.16 | 0.06 | -0.001 | 0.688 |
|  | Allergic rhinitis | 188.04 | 0.01 | >-0.001 | 0.042 |
|  | Allergic asthma | 175.39 | 0.04 | -0.002 | 0.758 |
| Insomnia | Atopic dermatitis | 53.36 | 0.04 | 0.004 | 0.539 |
|  | Allergic rhinitis | 46.1 | 0.15 | <0.001 | 0.796 |
|  | Allergic asthma | 39.69 | 0.35 | 0.011 | 0.367 |
