# Supplementary material for: Insomnia Associated With Increased Risk of Atopic Dermatitis: A Two‐Sample Mendelian Randomization Study
Source: Brain Behav. 2025 May 5;15(5):e70512. doi: 10.1002/brb3.70512 (PMC12050649; doi:10.1002/brb3.70512)
Supplement: Supplementary file 7 — Table S4. MR‐PRESSO test results for detecting and correcting outlier effects in Mendelian randomization analysis. [file BRB3-15-e70512-s003.docx]

**Table S4. MR-PRESSO test results.**

| **Exposure** | **Outcome** | **Raw** | | | **Outlier corrected** | | | **Global P** | **Number of outliers** | **Distortion P** |
| --- | --- | --- | --- | --- | --- | --- | --- | --- | --- | --- |
|  |  | **OR** | **95%CI** | **P** | **OR** | **95%CI** | **P** |  |  |  |
| Short sleep | Allergic rhinitis | 1.01 | 0.96-1.07 | 0.58 | 0.99 | 0.95-1.03 | 0.75 | <0.001 | 1 | 0.088 |
| Chronotype | Allergic rhinitis | 0.99 | 0.988-0.998 | 0.005 | 0.99 | 0.990-0.999 | 0.008 | <0.001 | 1 | 0.627 |
